# Comparison between Amulet and Watchman left atrial appendage closure devices: A real-world, single center experience

**DOI:** 10.1016/j.ijcha.2021.100893

**Published:** 2021-10-19

**Authors:** Mohammed Saad, Osama Risha, Makoto Sano, Thomas Fink, Christian-Hendrik Heeger, Julia Vogler, Vanessa Sciacca, Charlotte Eitel, Thomas Stiermaier, Alexander Joost, Ahmad Keelani, Georg Fuernau, Roza Meyer-Saraei, Karl-Heinz Kuck, Ingo Eitel, Roland Richard Tilz

**Affiliations:** a Medical Clinic III, University Hospital Schleswig-Holstein, Kiel, Germany; b Medical Clinic II, University Heart Center Lübeck, Lübeck, Germany; c Medical Clinic II, University Heart Center Lübeck, Division of Electrophysiology, Lübeck, Germany; d German Centre for Cardiovascular Research (DZHK), Partner Site Hamburg/Kiel/Lübeck, Lübeck, Germany

**Keywords:** Left atrial appendage closure, Amulet, Watchman, Atrial fibrillation, Anti-coagulation, ACP, Amplatzer Cardiac Plug, AF, Atrial fibrillation, LAA, Left atrial appendage, LAAC, Left atrial appendage closure, MACCE, major adverse cardiac and cerebrovascular events, OAC, Oral anticoagulants, TEE, transesophageal echocardiography

## Abstract

**Background:**

Data reporting a head-to-head comparison between Amulet and Watchman devices are scarce. The aim of this study was to compare the Watchman™ versus Amulet™ left atrial appendage closure (LAAC) devices in a consecutive, industry-independent registry.

**Methods:**

Patients who underwent LAAC using Watchman or Amulet devices from January 2014 to December 2019 at the University Heart Center Lübeck, Lübeck, Germany were included in the present analysis. Primary endpoints included periprocedural complications (in-hospital death, pericardial tamponade, device embolization, stroke, major bleeding and vascular access complications), and complications during long-term follow-up (ischemic stroke, hemorrhagic stroke, thromboembolism, device thrombus, bleeding and death).

**Results:**

After matching the patients for age (±5 years), gender, CHA2DS2Vasc score (±1) and HASBLED score (±1), each of the Watchman and the Amulet groups included 113 patients. Patients in the Amulet group had significantly more periprocedural complications (2.7% vs 10.6%, p = 0.029; respectively) and more major bleeding complications (0% vs 5.3%, p = 0.029; respectively). During long-term follow-up, the rate of events was comparable between the Watchman and Amulet groups (18.3% versus 20.8%, p = 0.729; respectively).

**Conclusion:**

Amulet LAAC device was associated with increased periprocedural complications as compared to Watchman LAAC device. On long-term follow-up, both devices showed comparable efficacy and safety.

## 1 Introduction

Atrial fibrillation (AF) is the most common form of clinically relevant arrhythmia [1] with a prevalence ranging from 0.1% among patients aged <55 years and up to 9% in octogenarians [2]. Patients with AF have an increased risk of ischemic stroke approximately 5 times compared with those without AF [3]. Although oral anticoagulants (OAC) play an important role in preventing AF-related stroke [4], [5], this treatment is underutilized in a subset of patients due to poor patient compliance, contraindications, and potential bleeding complications [6], [7], [8], [9], [10]. Taking into consideration that 90% of thrombi are located in the left atrial appendage (LAA) in patients with non-valvular AF [11], left atrial appendage closure (LAAC) has emerged as an alternative approach in this patient group.

Currently, many percutaneous LAAC devices have obtained CE mark. The Watchman (Boston Scientific, Marlborough, MA, USA) and the Amplatzer Cardiac Plug (ACP) (Abbott, St Paul, MN, USA) are the most commonly used devices for mechanical orifice obstruction and the Lariat device (SentreHEART, Redwood City, CA, USA) for epicardial suture ligation [12]. The Watchman device is approved in many countries worldwide and is the only device studied in randomized trials [13], [14], as well as in multicenter prospective non-randomized studies [15], [16]. For ACP and its second-generation Amulet, multiple retrospective and prospective registries have reported successful device implantation in 95–100% of patients, with a low rate of major periprocedural adverse events [17], [18], [19], [20], [21], [22]. A recently published, industry-initiated (Abbott), large, randomized, multi-center, trial (Amulet IDE trial) evaluating the safety and effectiveness of the Amulet occluder compared with the Watchman™ device, has shown that the Amulet occluder was non-inferior for safety and effectiveness of stroke prevention compared with the Watchman device [23]. Otherwise, data reporting a head-to-head comparison of clinical outcomes in a consecutive, industry-independent registry of Amplatzer and Watchman devices are limited [24], [25], [26], [27], [28], [29]. Aim of this study was therefore to compare the Watchman™ to the Amulet™ LAAC device regarding *peri*-procedural success and complications during long-term follow-up in a real-world industry-independent registry.

## 2 Methods

### 2.1 Study population

**Fig. 1:**
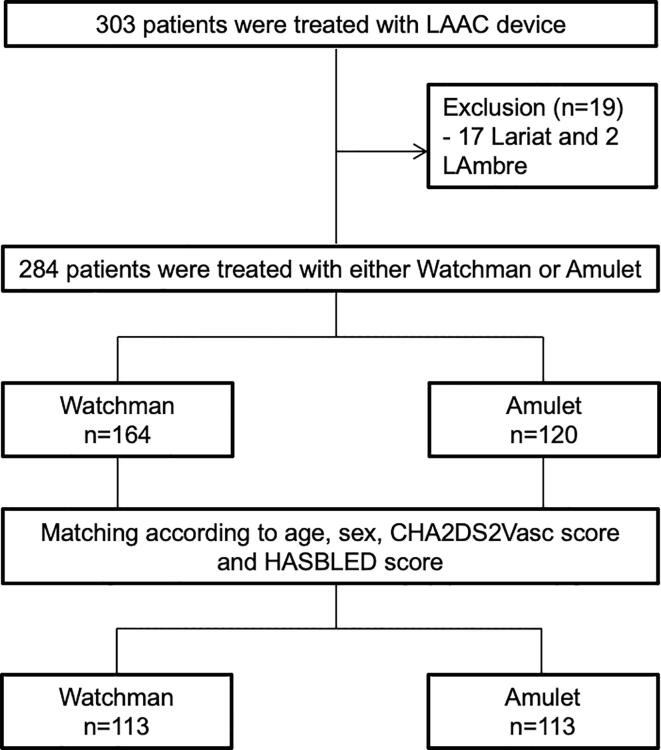
Study flow chart. LAAC: left atrial appendage closure.

Patients who underwent endocardial or epicardial LAAC from January 2014 to December 2019 at the University Heart Center Lübeck, Lübeck, Germany, were screened for this study. Patients who underwent LAAC using Watchman or Amulet devices were included in the present analysis. Exclusion criteria of the study included patients who were treated with LAAC devices other than Watchman and Amulet (Fig. 1). The implantation of LAAC devices were done by experienced and certified implanters and according to the recommendation of EHRA/EAPCI expert consensus statement for catheter-based LAAC [30].

Determination of device type were left to operator discretion according to anatomy of LAA and/or experience of the operator. Analysis was conducted retrospectively and data were anonymized for analysis. To adjust for baseline differences between the 2 groups, patients were matched for age (±5 years), gender, CHA2DS2Vasc score (±1) and HASBLED score (±1) (Fig. 1). The primary endpoints of the study included the occurrence of *peri*-procedural complications and complications during long-term follow-up. The study was conducted in accordance with the Declaration of Helsinki and was approved by the local ethics board. All patients provided informed consent to the procedure before intervention.

### 2.2 Definition of endpoints

Procedural endpoints and adverse events were categorized according to the Munich consensus document on LAAC [31]. Periprocedural complications included in-hospital death, pericardial tamponade, device embolization, stroke, major bleeding and vascular access complications. Complications during long-term follow-up included ischemic stroke, hemorrhagic stroke, thromboembolism, device thrombus, bleeding and death. Endpoints were analyzed individually as well as in combination as major adverse cardiac and cerebrovascular events (MACCE).

### 2.3 Periprocedural management of LAAC

All patients underwent preprocedural transthoracic and transesophageal echocardiography (TEE) to measure left ventricular ejection fraction and to rule out left atrial thrombi. No assessment of LAA anatomy was done using CT. All procedures were performed under deep sedation using midazolam, fentanyl and continuous infusion of propofol. Intravenous heparin was administered to maintain an activated clotting time of >300 s throughout the procedure. Operators were either electrophysiologists or interventional cardiologists, depending on the indication for LAAC. Transseptal puncture was performed under fluoroscopic and/or TEE guidance using an SL1 sheath. Electrophysiologist performed the transseptal puncture mainly fluoroscopic guided while interventional cardiologists TEE-guided. Implantation of LAAC device is done for all cases under fluoroscopy and TEE guidance. In patients with prior pulmonary vein isolation procedure with or without LAA electrical isolation, angiography of the pulmonary veins was performed. Reconduction of the pulmonary veins was assessed using a decapolar circular mapping catheter (Lasso, Biosense Webster) before deploying the LAAC device. The device-suitable sheath was inserted into the left atrium over a wire. A pigtail catheter was positioned into the LAA over a soft J-tipped 0.035-inch wire for angiography and for advancing the sheath into the LAA. Optimal device type and size was determined after echocardiographic and angiographic visualization and determination of the shape, number of lobes and diameter of ostium (Watchman), landing zone (Amulet) and depth of the LAA. Measurements were done in angiography in right anterior oblique (RAO) caudal and cranial projections and in TEE in 0°, 45°, 90° and 135°. The device size is typically chosen 10–20% larger than the diameter of the ostium/landing zone and the device was deployed under fluoroscopy and TEE guidance. Complete LAA closure was assessed by TEE and LAA-angiography after closure. A tug test was performed under fluoroscopy or TEE demonstrating simultaneous movement of the device and appendage and finally the device was released after confirmation of optimal positioning.

### 2.4 Post-procedural management and follow-up visits

All patients underwent transthoracic echocardiography to rule out pericardial effusion and device migration at the first post-procedural day. Post procedural antithrombotic therapy was prescribed according to physician’s discretion and based on individual patient characteristics, presence of indications for anti-platelet therapy, indications for OAC besides underlying AF, and history of bleeding events. OAC or dual anti-platelet therapy was usually continued until successful LAAC was confirmed by the next follow-up TEE. In patients with endocardial LAAC and absence of a mandatory indication for OAC, dual or single anti-platelet therapy was prescribed. In patients undergoing AF ablation procedure within the previous 2 months, novel oral anticoagulation therapy was typically prescribed. Follow-up visits with planned TEE were scheduled after 6 to 12 weeks after the procedure to evaluate *peri*-device leakage and device thrombosis and to evaluate the occurrence of other adverse events. Afterwards, clinical scheduled follow-up was regularly carried out every 6–12 months at the outpatient clinic or the referring clinic. Mortality was documented based on hospital visits, scheduled follow-up visits and communication with ambulatory physicians.

### 2.5 Statistical analysis

To adjust for baseline differences between the Watchman and Amulet group, patients were matched for age (±5 years), gender, CHA2DS2Vasc score (±1) and HASBLED score (±1). Categorical data were presented as frequency with percentage. Continuous data were expressed as median with interquartile range. Differences between groups were assessed by Fisher’s exact or the *X*^2^ test for categorical variables, and were evaluated using the nonparametric Mann-Whitney *U* test for continuous data. Kaplan-Meier graphs were used to illustrate the complication rates during long-term follow-up in the two groups. All tests were 2-tailed, and a p-value < 0.05 was considered statistically significant. Statistical analysis was performed using SPSS Statistics 17.0.0.0 (IBM, Armonk, New York).

## 3 Results

### 3.1 Patient characteristics

From January 2014 to December 2019, 303 patients underwent endocardial or epicardial LAAC at the University Heart Center Lübeck, Lübeck, Germany. Of these, 284 patients underwent LAAC using Watchman or Amulet devices and were included in the present analysis. Patients who underwent epicardial LAAC with Lariat (17 patients) or endocardial LAAC using Lambre (2 patients) devices were excluded from this study.

After matching the patients for age (±5 years), gender, CHA2DS2Vasc score (±1) and HASBLED score (±1), 113 patient pairs with 113 patients in Watchman group were compared to 113 patients in the Amulet group (Fig. 1). Median follow-up was 238 days in the Watchman group and 160 days in the Amulet group. Patients in the Amulet group included more patients with a history of congestive heart failure and more patients with a history of hemorrhagic stroke. Otherwise, there were no significant differences in patient characteristics between both groups (Table 1). The procedural device implantation success was achieved in 99% in the Watchman group and in 97% in the Amulet group.

**Table 1:** Baseline and procedural characteristics.

**Variable**	**Watchman** (n = 113)	**Amulet** (n = 113)	***P***
Age (years)	77 (72–82)	78 (74–82)	0.91
Male sex	70/113 (62%)	70/113 (62%)	1.000
Hypertension	100/113 (89%)	101/113 (89%)	1.000
Diabetes mellitus	35/113 (31%)	37/113 (33%)	0.887
Body mass index (kg/m^2^)	26 (24–30)	26 (24–28)	0.310
Ischemic heart disease	53/109 (49%)	55/112 (49%)	1.000
Congestive heart failure	29/113 (26%)	44/113 (39%)	0.046
Peripheral vascular disease	40/113 (35%)	41/113 (36%)	1.000
Liver Dysfunction	2/113 (2%)	5/113 (4%)	0.446
History of Ischemic stroke	22/113 (20%)	19/113 (17%)	0.730
History of TIA	10/113 (9%)	7/113 (6%)	0.615
History of hemorrhagic stroke	43/113 (38%)	69/113 (61%)	0.001
History of major bleeding	56/113 (50%)	65/113 (58%)	0.286
LVEF	55 (45–55)	53 (45–55)	0.460
NYHA Class			0.127
1	81/107 (76%)	69/112 (62%)	
2	9/107 (8%)	19/112 (17%)	
3	17/107 (16%)	23/112 (20%)	
4	0/107 (0%)	1/112 (1%)	
CHA2DS2VASC-SCORE	4 (3–5)	4 (3–5)	1.000
HASBLED-SCORE	3 (2–3)	3 (2–3)	0.890
LAA-flow	40 (27–69)	40 (24–60)	0.421
Serum Creatinin	102 (86–128)	98 (80–134)	0.751
GFR	59 (48–71)	58 (40–73)	0.348
Anticoagulation before implantation			0.176
No	5/105 (4.8%)	2/112 (1.8%)	
SAPT	6/105 (5.7%)	7/112 (6.3%)	
DAPT	8/105 (7.6%)	10/112 (8.9%)	
NOAC + Clopidogrel	6/105 (5.7%)	7/112 (6.3%)	
NOAC	42/105 (40%)	60/112 (53.6%)	
VKA	35/105 (33.3%)	26/112 (23.2%)	
Triple	3/105 (2.9%)	0/112 (0%)	
Anticoagulation after implantation			0.003
Unknown	10/112 (8.9%)	0/112 (0%)	
ASS	1/112 (0.9%)	0/112 (0%)	
Clopidogrel	0/112 (0%)	1/112 (0.9%)	
DAPT	68/112 (60.7%)	89/112 (79.5%)	
NOAC + Clopidogrel	3/112 (2.7%)	1/112 (0.9%)	
NOAC	18/112 (16.1%)	12/112 (10.7%)	
VKA	12/112 (10.7%)	6/112 (5.4%)	
Triple	0/112 (0%)	3/112 (2.7%)	
Hospital stay (days)	4 (3–7)	6 (3–12)	0.713
Device size (mm)	27 (24–27)	25 (22–28)	0.118
Contrast volume (ml)	80 (50–105)	50 (40–95)	0.003
Fluoroscopy time (minutes)	9 (7–13)	10 (7–14)	0.290
Radiation dose	2450 (1464–3609)	1650 (1222–2896)	0.655
Implantation success	112/113 (99%)	110/113 (97%)	0.247
Major Leak (>5 mm)	1/113 (1%)	0/113 (0%)	1.000
Minor Leak (<5 mm)	6/113 (5%)	2/113 (2%)	0.280
Follow-up duration (days)	238 (99–621)	160 (75–379)	0.01

Continuous data are presented as median and interquartile range

DAPT = dual antiplatelet therapy; GFR = glomerular filtration rate; LAA = left atrial appendage; LVEF = left ventricular ejection fraction; NOAC = new oral anticoagulants; NYHA = New York heart association; SAPT = single antiplatelet therapy; TIA = transient ischemic attack; VKA = vitamin K antagonists.

### 3.2 Periprocedural complications

In comparison to patients in the Watchman group, patients in the Amulet group had significantly more periprocedural complications (2.7% vs 10.6%, p = 0.029; respectively) and more major bleeding complications (0% vs 5.3%, p = 0.029; respectively). The detailed events are illustrated in Table 2. In-hospital death, pericardial tamponade and device embolization were as well numerically higher in the Amulet group but without reaching statistical significance (Table 2).

**Table 2:** Complications.

**Variable**	**Watchman**	**Amulet**	***p***
*Procedural complications*			
MACCE – n (%)	3/113 (2.7)	12/113 (10.6)	**0.029**
Minor complications – n (%)	2/113 (1.8)	1/113 (0.9)	1.000
In-hospital death – n (%)	1/113 (0.9)	4/113 (3.5)	0.369
Pericardial tamponade – n (%)	0/113 (0.0)	5/113 (4.4)	0.060
Device embolization – n (%)	0/113 (0.0)	4/113 (3.5)	0.122
Stroke – n (%)	1/113 (0.9)	0/113 (0.0)	1.000
Major bleeding – n (%)	0/113 (0,0)	6/113 (5.3)	**0.029**
Blood transfusion – n (%)	2/113 (1.8)	5/113 (4.4)	0.446
Major vascular access complications	1/113 (0.9)	2/113 (1.8)	1.000
Minor vascular access complications	2/113 (1.8)	1/113 (0.9)	1.000
*Complications during long-term follow-up*			
MACCE – n (%)	19/104 (18.3)	22/106 (20.8)	0.729
Ischemic stroke – n (%)	3/104 (2.9)	2/106 (1.9)	0.681
Hemorrhagic Stroke – n (%)	0/104 (0.0)	2/106 (1.9)	0.498
Thromboembolism – n (%)	2/104 (1.9)	2/106 (1.9)	1.000
Device-Thrombus – n (%)	5/102 (4.9)	1/106 (0.9)	0.114
Bleeding – n (%)	9/104 (8.7)	9/106 (8.5)	1.000
Death – n (%)	10/104 (9.6)	15/106 (14.2)	0.395
Cardiac death – n (%)	1/104 (1.0)	4/106 (3.8)	0.342

MACCE = major adverse cerebral and cardiovascular events.

### 3.3 Clinical outcome during long-term follow-up

**Fig. 2:**
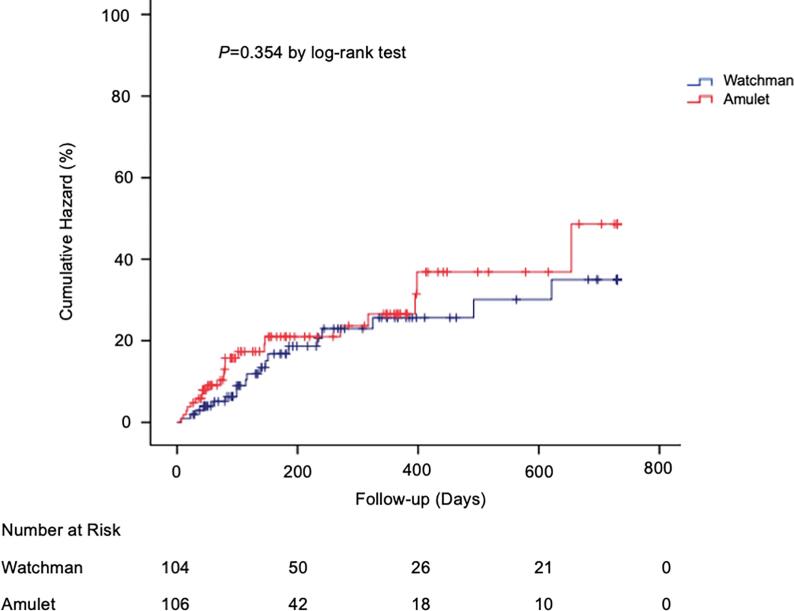
Kaplan-Meier graph showing long-term follow-up of cumulative major adverse cardiovascular and cerebral event rates according to Watchman and Amulet left atrial appendage colure devices.

Anticoagulation strategy at the end of the follow up is shown in Table 1. Most of the patients were discharged on dual-antiplatelet-therapy in both groups (60.7% in Watchman group and 79.5% in Amulet group) followed by OAK or new OAK (10.7% and 16.1% for Watchman and 5.4% and 10.7% for Amulet, respectively). Otherwise, very few patients were discharged on single antiplatelet-, triple- or new OAC/Clopidogrel-therapy. During long-term follow-up, the rate of MACCE was similar between the 2 groups. However, the rate of developing a device-thrombus was numerically higher in the Watchman group (4.9%) in comparison to the Amulet group (0.9%), but it did not reach statistical significance (Table 2). Fig. 2 depicts Kaplan–Meier plots showing the risk of MACCE in both groups during long-term follow-up with no relevant difference between groups (p = 0.354).

## 4 Discussion

In our consecutive, real-world, industry-independent registry study comparing Watchman versus Amulet LAAC device implantation, the implantation of Amulet LAAC device was associated with an increased risk for periprocedural complications without increase in complication rates during long-term follow-up.

In the previously published trials and registries of both the Amplatzer devices (ACP and Amulet) and the Watchman device, both devices proved clinical effectiveness in prevention of ischemic and bleeding events, as well as cardiovascular mortality [13], [14], [15], [16], [32], [33], [34]. In the first large scale randomized trial to assess the Amulet occlude compared with the Watchman device (Amulet IDE trial), the Amulet occluder was non-inferior for safety and effectiveness of stroke prevention compared with the Watchman device [23]. Apart from the Amulet IDE trial, only few retrospective data reported a head-to-head comparison of clinical outcomes of Amplatzer and Watchman devices and especially industry-independent data are lacking [24], [25], [26], [27], [28], [29].

In our real-world study, the incidence rate of periprocedural MACCE for our cohort was 6.6%, with an incidence of 2.7% in the Watchman group and 10.6% in the Amulet group. In the previously published Watchman studies, the rates of 7-day procedure-related serious adverse events were 8.7% in PROTECT-AF trial, 4.2% in the PREVAIL trial, and 2.8% in the EWOLUTION study [13], [14], [15]. However, we reported a higher incidence of MACCE in the Amulet group than in previously published large registries with rates ranging from 3.2% to 6.2% [17], [32], [34].

Consistent with our results, procedure-related complications in the randomized Amulet IDE trial were higher for the Amulet occluder (4.5% vs. 2.5% for Watchman), which were largely related to more frequent pericardial effusion and device embolization. Again, we had a numerically higher complication rates in the Amulet group than that in the Amulet IDE trial [23]. On the other hand, in the previously published data comparing Watchman and Amulet LAAC devices, the rate of overall major *peri*-procedural complications did not differ between Watchman and Amplatzer groups [24], [25], [26], [27], [28], [29]. This may be explained by the small number of patients included in these retrospective studies, lack of randomization or matching between the 2 device groups or both reasons together [25], [26], [27], [28], [29]. The significantly higher incidence of periprocedural complications associated with Amulet implantation in our matched patient populations was mainly derived from the numerically higher incidence of pericardial tamponade, device embolization and major bleeding. These differences may be attributable to the early implantation experience with the Watchman device in our center and the introduction of the Amulet device later at the end of 2016 or may be explained with a selection bias.

In our analysis, there were 4 (3.5%) device embolizations in the Amulet group with no documented embolization in the Watchman group. Three embolizations occurred directly after the implantation procedure while the fourth occurred few hours later and all were treated percutaneously with no need for a surgical retrieval. One embolization was due to an oversized device, while the second was due to an undersized device and both were retrieved from the left atrium. The third embolization was due to a difficult LAA anatomy (broccoli LAA), whereas the fourth device dislodged from its delivery catheter and was partially embolized in the left atrium before its implantation in the LAA followed by complete embolization in the inferior vena cava during a trial of retrieval.

In a propensity score matched analysis of 2 real-world registries, higher rates of device embolizations, bailout surgery, and cardio-pulmonary resuscitation were noticed in the Amplatzer group compared to the Watchman group. In these 2 real-world registries, 8 (3%) device embolizations occurred in patients who received the Amplatzer devices and only 1 embolization in patients who received the Watchman device [24]. However, the rate of device embolization was much lower (0.7%) in the previously published LAAC observational studies of AMPLATZER devices [32], [34]. Again, in the Amulet IDE trial, the higher incidence of procedure complications in the Amulet group were largely related to more frequent pericardial effusion and device embolization [23].

We reported a significantly higher incidence of major bleeding in the Amulet group than in the Watchman group (5.3% vs 0% respectively, p = 0.029) and a numerically higher incidence of pericardial tamponade (4.4% vs 0% respectively, p = 0.06). Similarly, in a total of 371 consecutive patients from 8 UK centers, Betts et al reported a significantly higher incidence of acute minor adverse events in patients who were treated with ACP compared to patients who were treated with Watchman and this was explained with the higher number of incidental pericardial effusions and bleeds [26]. In the recently published subanalysis of the Left-Atrium-Appendage Occluder Register - GErmany (LAARGE) registry, procedural safety did not differ between Watchman and Amulet devices. However, the incidence of severe bleeding and moderate pericardial effusion were numerically higher in the Amulet group without reaching a statistical significance and the incidence of moderate bleeding was significantly higher in the Amulet group [35]. While the incidence rate of periprocedural vascular access complications was similar between the 2 groups in our study, other studies reported higher rates in patients who were treated either with Watchman [24] or with Amulet [28]. This might be explained in part by the different implantation settings in the different centers either with using a radial, femoral or no arterial access in monitoring blood pressure during LAAC device implantation and in part the heterogenous use of vascular closure techniques.

In our study, procedural radiation doses and fluoroscopy times were similar for the two devices, while the dose of contrast was higher for Watchman compared to Amulet. Figini et al reported comparable results for intraprocedural radiation doses and fluoroscopy times but higher volume of contrast for ACP [25].

In the present report, Watchman and Amplatzer devices offered comparable efficacy and safety on long-term follow-up in patients with non-valvular atrial fibrillation. No statistically significant differences were found in terms of deaths (total and cardiac), thromboembolic and bleeding events at follow-up. These results are consistent with the previously published reports comparing Watchman and Amulet LAAC devices [23], [24].

The stroke rate at follow-up was 2.9% in patients who were treated with Watchman and 1.9% in patients who were treated with Amulet consistent with other reports of Amplatzer registries [17], [32], [34] and in the 5-year outcomes of the PROTECT-AF and PREVAIL trials for the Watchman occluder [36].

The incidence rate of device-thrombus with the Watchman device was 4.9%, which is relatively consistent with rates observed in the PROTECT AF and ASAP (ASA Plavix Feasibility Study with Watchman Left Atrial Appendage Closure Technology) studies [13], [16], which were 4.2% and 4%, respectively. In the Amulet group, we reported an incidence of only 0.9% of device thrombus, a rate that is lower than the 2–4% reported in the Amplatzer registries [19], [32], [34]. Fauchier et al reported an overall incidence of thrombus on the device of 5.5% in patients treated with a Watchman device, 8.2% with the ACP, and 25% with the Amulet device [27].

We noticed a numerically higher incidence of *peri*-device leak <5 mm in the Watchman group (5%) than in the Amulet group (2%), but without reaching a statistical significance. In the Amulet IDE trial, successful device-based LAA occlusion with residual jet around the device ≤5 mm was observed in 98.9% of Amulet patients and 96.8% of Watchman patients (p = 0.003) [23]. However, in the matched analysis of Kleinecke et al, *peri*-device leak ≥5 mm was equally low for Amplatzer and Watchman groups [24]. In the study of Figini et al, and during follow-up, there was a significantly higher incidence of severe *peri*-device leak (>3 mm) with the Watchman device [25].

We reported a death rate of 9.6% in the Watchman group and 14.2% in the Amulet group. These mortality rates are higher than in other studies [13], [14], [25], [27], [28], [29]. In comparison to patients of the PROTECT-AF, CAP and PREVAIL studies, our patients were older, had more prevalence of diabetes, congestive heart failure and higher incidence of transient ischemic attack, ischemic and hemorrhagic stroke [14]. At 1-year follow-up in the EWOLUTION registry, the mortality rate was 9.8%, and this was mainly attributed to the advanced age and considerable comorbidities of the study patients [15]. In comparison to the EWOLUTION registry, our patients have a significantly higher prevalence of hemorrhagic stroke and major bleeding [37]. Moreover, in a matched analysis of Kleinecke et al, all-cause mortality at 1 year for Watchman was 8.36% (54/646) and for Amulet 10.21% (69/676); a rate which is close to our results [24].

Despite most of the retrospective data showing comparable efficacy and safety of the Watchman and Amplatzer devices, larger randomized studies or well-designed prospective registries will have to confirm these results. CLOSURE-AF (NCT03463317) trial is the largest on-going, non-industry sponsored, trial comparing LAAC to oral anticoagulants. Results of this study are expected to give more information about the safety and efficacy of LAAC in a real-world patient population.

### 4.1 Limitations

Due to lack of randomization, potential selection bias cannot be excluded. However, to minimize this potential bias, we performed a matching for age, gender, CHA2DS2Vasc score, and HASBLED score for the 2 groups. Furthermore, we started earlier in our center with the implantation of the Watchman device, which may have influenced the operator experience, *peri*-procedural complications and the significantly different follow-up durations. Other limitations of this study include the small number of patients, selection of the LAAC device according to the operator decision and expertise and different post-interventional anti-coagulations strategies over the time of study period.

## 5 Conclusion

Our results of a large, industry-independent real-world registry demonstrate that in patients with non-valvular atrial fibrillation undergoing LAAC, the implantation of Amulet LAAC device was associated with increased periprocedural complications compared to Watchman LAAC device. Both devices showed comparable efficacy and safety on long-term follow-up.

## 6 Author responsibility

All authors take responsibility for all aspects of the reliability and freedom from bias of the data presented and their discussed interpretation.

## 7 Grant support

None.

## Declaration of Competing Interest

The authors declare the following financial interests/personal relationships which may be considered as potential competing interests: RRT: consultant to Boston Scientific, speaker‘s bureau: Boston Scientific, Abbot Medical. MS: proctor in Boston Scientific for Watchman 2.5 and Watchman FLX. All other authors declare no competing financial and/or non-financial interests in relation to the work described.
